# Supplementary material for: Acute Oral Toxicity and Genotoxicity Test and Evaluation of Cinnamomum camphora Seed Kernel Oil
Source: Foods. 2023 Jan 8;12(2):293. doi: 10.3390/foods12020293 (PMC9857420; doi:10.3390/foods12020293)
Supplement: Supplementary file 1 [file foods-12-00293-s001.zip › foods-2119863-supplementary.pdf]

foods

Supplementary Information

1. Materials

1.1. Tested substance

The tested substance, CCSKO, was produced from the *Cinnamomum camphora* seeds (CCS) through the following processes. Firstly, CCS kernels were produced by removing the pericarp of CCS, rubbing and washing with peeling machine, grading based on particle size difference with vibration screening machine, breaking the testae of the seed cores with three roll pressing shellers, and removing the broken testae based on density and color difference with the kernel and testa separator. Then, the crude CCSKO was extracted from CCSK by hot-pressing (HP) method and aqueous enzyme extraction (AEE) method, respectively.

HP: The oil obtained by hot-pressing method was according to Siger et al. [1] with slight modifications. The CCS kernels were pressed at room temperature using an oil pressing machine (Farmet UNO 1F, Farmet a.s., Česká Skalice, Prague, Czech Republic). The temperature inside the machine was 160 ± 10 °C. Once produced, the oil was centrifuged at 4000 rpm for 15 min, and stored at 4 °C in the dark.

AEE: The neutral protease solution was produced by *Bacillus amyloliticus* NCU116-1 fermentation for 4 hours (China patent number: ZL201510280502.9). The CCSK were emulsified with water with the ratio of 1:4 (w/w) in colloid grinder, and the emulsion was hydrolyzed and broken by neutral protease (derived from *B. amyloliquefaciens* NCU116-1) with the ratio of 1:5 (v/v) in a refining tank. The crude oil was obtained by centrifugation at 4000 *g*, 4 °C for 30 min, and stored at 4 °C in the dark [2].

1.2. Chemical reagents

1.2.1. Chemical reagents in mammalian erythrocyte micronucleus test

Calf serum was purchased from Beijing Solarbio Science & Technology Co. Ltd. (Beijing, China). Giemsa stain, phosphate buffer solution (PBS) and methanol were purchased from Shanghai aladdin Biochemical Technology Co.,Ltd. (Shanghai, China).

1.2.2. Chemical reagents in bacterial reverse mutation test (Ames test)

Beef extract powder, tryptone, sodium chloride (NaCl), dipotassium phosphate ($K_2HPO_4{\cdot}3H_2O$), agar powder, glucose and DMSO were purchased from Shanghai aladdin Biochemical Technology Co., Ltd. (Shanghai, China). Vogel-Bonner medium E, D-biotin, L-histidine, ampicillin, crystal violet and tetracycline were purchased from Avantor Inc (Radnor, PA, USA). S9 (prepared in our laboratory), stored in the refrigerator at -80 °C for later use.

1.2.3. Chemical reagents in *in vitro* mammalian cell TK gene mutation test

RPMI1640 medium was purchased from Thermofisher Scientific Inc (Massachusetts, USA). Horse serum was purchased from Beijing Solarbio Science & Technology Co. Ltd. (Beijing, China). Oxidized coenzyme II, sodium glucose 6-phosphate, methyl mesylate (MMS), cyclophosphamide (CP), trifluorothymidine (TFT), methotrexate (M), thymidine (T), hypoxanthine (H) and glycine (G) were purchased from Avantor Inc (Pennsylvania, USA). S9 (prepared in our laboratory), stored in the refrigerator at -80 °C for later use.

1.3. Instruments and equipment

1.3.1. Instruments and equipment in mammalian erythrocyte micronucleus test

The instruments and equipment used were as follows: mouse surgical kits (Kent Scientific Co. Ltd., Torrington, CT, USA) and biomicroscope (Carl Zeiss AG Co. Ltd., Baden-Württemberg, Germany).

1.3.2. Instruments and equipment in bacterial reverse mutation test (Ames test)

The instruments and equipment used were as follows: Sorvall ST 16R Centrifuge (Thermo Fisher Scientific Inc, Waltham, MA, USA), cryogenic refrigerator (-80 °C) (Thermo Fisher Scientific Inc, Waltham, MA, USA), biological safety cabinet (Thermo Fisher Scientific Inc, Waltham, MA, USA), 2102C constant temperature oscillator (Zhicheng Inc., Shanghai, China), thermostatic water bath (Jinghong Inc., Shanghai, China), C1109C sterilizing installation (Zhicheng Inc., Shanghai, China), T18 brushless digital homogenizer (IKA Works Guangzhou & Co., Guangzhou, China) and other laboratory commonly used instruments and equipment.

1.3.3. Instruments and equipment in *in vitro* mammalian cell TK gene mutation test

The instruments and equipment used were as follows: Sorvall ST 16R Centrifuge (Thermo Fisher Scientific Inc, Waltham, MA, USA), cryogenic refrigerator (-80 °C) (Thermo Fisher Scientific Inc, Waltham, MA, USA), biological safety cabinet (Thermo Fisher Scientific Inc, Waltham, MA, USA), SKP-02.420 thermostatic incubator (Huangshi Hengfeng Medical Instrument Co. Ltd., Huangshi, China), biomicroscope (Carl Zeiss AG Co. Ltd., Baden-Württemberg, Germany) and other laboratory commonly used instruments and equipment.

2. Test standards and principles

2.1. Acute oral toxicology test

Acute oral toxicology test was conducted according to the OECD Guideline for Testing of Chemicals 423 (Horn's method) [3] and the National Food Safety Standard of China - acute oral toxicology test (GB 15193.3-2014) (Horn's method).

Acute oral toxicity tests can provide information on the health hazards caused by oral exposure to test substances in a short period of time, thus provide the basis for the dose selection of further toxicity tests, and preliminarily estimate the target organs and possible mechanisms of toxicity.

2.2. Mammalian erythrocyte micronucleus test

Mammalian erythrocyte micronucleus test was conducted according to the OECD Guideline for Testing of Chemicals 474 (30 h oral gavage infection method) [4] and the national food safety standard of China - mammalian erythrocyte micronucleus test (GB 15193.5-2014).

Mammalian erythrocyte micronucleus test is an *in vivo* test with short period. In this test, mice bone marrow and/or peripheral blood cells are analyzed to investigate the impairment of mature erythrocyte chromosomes or mitotic apparatus by subjects, which lead to generate flat-footed chromosome fragments or micronucleus in whole chromosome. The result is mainly considered to the affection of chromosome clastogen, furthermore, a group of smaller nuclei act as alternative to the unformed major nucleus in the influence of spindle toxicant, can also thought as the reason.

2.3. Bacterial reverse mutation test (Ames test)

Bacterial reverse mutation test was conducted by the OECD Guideline for Testing of Chemicals 471 (Plate infiltration method) [5] and the national food safety standard of China- bacterial reverse mutation test (GB 15193.4-2014).

Bacterial reverse mutation (Ames) assay is aimed to observe the gene mutant affection of microbes (especially bacteria), predict the hereditary toxicity and potential carcinogenic affection successively. *Salmonella typhimurium* (*S. typhimurium*) and/or *Escherichia coli* (*E. coli*) are selected to detect point mutation, including the substitution, insertion and/or deletion of one or more DNA base pairs. *S. typhimurium* and *E. coli* bacterial strains are selected as histidine deficient and tryptophan deficient mutants, respectively. When

mutagenic material exists, they can revert to the prototrophic bacteria and grow normally on the basis free of histidine and/or tryptophan.

2.4. In vitro mammalian cell TK gene mutation test of CCSKO

In vitro mammalian cell TK gene mutation test (mouse lyphoma TK assay: MLA assay) was conducted according to OECD Guideline for Testing of Chemicals 490 [6] and national food safety standard of China (GB-15193.20-2014).

In vitro mammalian cell TK gene mutation is considered as the detective endpoint of *in vitro* mammalian cell TK gene mutation, which is classified as autosomal gene mutation. Generally, it is not an essential reaction, because thymidine-5′-monophosphoric acid (TMP) synthetase catalyzes deoxyuracil-5′-monophosphoric acid (dUMP) methylation to form TMP. However, when thymidine analogue, i.e., trifluorothymidine (TFT), is added in cell cultures, it embeds in DNA and causes lethal mutation. Therefore, mutagenic cells can grow normally in the basis with TFT, that is, presents resistance against TFT. Mutation frequency can be calculated according to the number of mutagenic colonies including large/small colonies (L5178Y cells) or normally growing/sluggishly growing colonies (TK6 cells), and the mutagenicity of subjects is inferred.

3. Methods

3.1. Determination of fatty acid composition of CCSKO

The fatty acid composition of CCSKO was determined according to the AOCS official method Ce 2-66 and AOCS official method Ce 1-62 [7, 8] and national food safety standard (GB 5009.168-2016), with slight modification. Methyl esterification of CCSKO was analyzed by an Agilent 7890B gas chromatograph with a DB-23 column (60 m × 250 μm × 0.25 μm, Agilent, USA). The major parameters were set as follows: carrier gas, nitrogen; flow rate, 2 mL/min; injection temperature, 250 °C, detection temperature, 250 °C. The temperature was firstly kept at 50 °C for 1 min, increased to 175 °C at 20 °C/min and kept at 175 °C for 5 min, increased from to 230 °C at 4 °C/min, and then kept at 230 °C for 10 min. The fatty acid profiles of CCSKO were obtained by comparing the gas chromatograms of CCSKO with 37 fatty acid methyl esters mixed standards (Anpel, China) using normalization method.

The *sn*-2 fatty acid composition of CCSKO was determined using a previous method [9], with slight modification. Pancreatic lipase (porcine pancreatic lipase, 15 mg), Tris buffer (pH 8.0, 10 mL), bile salts (0.05%, 2 mL) and calcium chloride (2%, 1 mL) were added to a test tube containing 10 mg CCSKO. The mixture was incubated in a water bath (37 °C) for 7 min with shaking. Then diethyl ether (5 mL) was added to stop the enzymatic reaction. Di-ethyl ether was absorbed by anhydrous sodium sulfate column, and evaporated by nitrogen gas. The hydrolytic product was separated on a silica gel G TLC plate, and the developing solvents were hexane/diethyl ether/acetic (70:30:1, v/v/v). The band corresponding to 2-monoacylglycerol (2-MAG) was scrapped off and extracted with diethyl ether (4 mL). The solvent was removed by anhydrous sodium sulfate column, and evaporated by nitrogen gas, and the residue was methylated and analyzed by gas chromatography (GC).

3.2. Determination of triglyceride composition of CCSKO

3.2.1. Quantitative analysis

The triglyceride composition of CCSKO was quantitatively analysed by a Agilent 1260 high performance liquid chromatograph (HPLC) (Agilent 1260, Agilent Technologies Inc., Santa Clara, CA, USA) equipped with evaporative light-scattering detector (ELSD) and an Elite C18 column (4.6 mm × 200 mm, 5 μm) (Thermo Fisher Scientific Inc, Waltham, MA, USA) according to the AOCS official method Ce 5b-89 [10], with slight modification. The flowing phases were composed of acetonitrile (A) and isopropanol (B) The major

parameters were set as follows: column temperature, 30 °C; injection volume, 5 μL; atomizing chamber temperature, 45 °C; nitrogen flow rate, 1.6 L/min. A linear gradient was carried out using a two-buffer gradient system, 70% A and 30% B at 0 min, the gradient became 50% A and 50% B in 30 min with the flow rate of 0.8 mL/min.

3.2.2. Qualitative analysis

The triglyceride composition of CCSKO was qualitatively analysed by an Agilent 6430 QqQ mass spectrometer equipped with an ESI source (Agilent technologies, Wilmington, USA). HPLC separation conditions were the same as those in Section **3.2.1**. The mass spectrometry analysis was conducted with atmospheric pressure chemical ionization (APCI) source under positive ion mode with the following conditions: drying temperature, 300 °C; dry gas flow, 5 L/min; nebulizer pressure 2.07 × 10^5 Pa; sheath gas temperature, 350 °C, sheath gas flow rate, 11 L/min; capillary voltage, 4.2 kV; shattering voltage, 150 V; collision energy, 25 V; mass scanning range, 100-1000 m/z.

3.3. Thermal behavior of CCSKO

The Fourier transform infrared spectroscopy (FTIR) spectrum of CCSKO was determined using a previous method [11]. Mid-infrared spectra (4000-400 cm^{-1}) were obtained by an FTIR spectrometer (Nicolet iS10, Thermo Nicolet Co., Waltham, MA, USA) with a deuterated tri-glycine sulphate detector (DTGS). The spectrometer was equipped with premium HATR ZnSe 45° trough plate accessory (HATR). Each spectrum was scanned 64 times with a resolution of 4 cm^{-1} and scan speed of 1 cm/s.

The Differential scanningcalorimetry (DSC) of CCSKO were determined using a DSC 8000 (Perkin Elmer Co. Ltd., Waltham, MA, USA) using a previous method [12] with some modifications. Briefly, CCSKO was accurately weighted and hermetically sealed in an aluminum sample pan, followed by heating from 17 °C to 50 °C in a rate of 50 °C/min. The thermogram was analysed by Pyris software (Perkin Elmer, Co. Ltd., Waltham, MA USA).

3.4. Determination of physicochemical properties of CCSKO

3.4.1. Physical properties

The relative density, refractive index, transparency, color, odor, heating test, moisture and volatile matter, insoluble impurity and residual solvent content of CCSKO were determined according to National Food Safety Standards (GB/T 5526-1985, GB/T 5529-1985, GB/T 5009.37-2003, GB/T 5525-2008, GB/T 5527-2010, GB 5009.262-2016, GB 5009.236-2016 and GB/T 5531-2018) and AOCS official method Cc 7-25 [13], AOCS official method Cc 13b-45 [14] and AOCS official method Ca 2f-93 [15] respectively.

3.4.2. Chemical properties

The acid value, unsaponifiable matter, saponification value, iodine value, peroxide value, arsenic, plumbum, benzopyrene (α) and aflatoxins B1 of CCSKO were determined according to AOCS official method Cd 3d-63 [16], National Food Safety Standards (GB 5009.229-2016, GB 5535.1-2008), AOCS official method Cd 3-25 [17], national food safety standard (GB/T 5534-2008), AOCS official method Cd 1b-87 [18] and national food safety standard (GB 5009.267-2020), AOCS official method Cd 8b-90 [19] and national food safety standard (GB 5009.227-2016), national food safety standard (GB 5009.11-2014), national food safety standard (GB 5009.12-2017), national food safety standard (GB 5009.27-2016), AOCS recommended practice Aa 11-05 [20] and national food safety standard (GB 5009.22-2016), respectively.

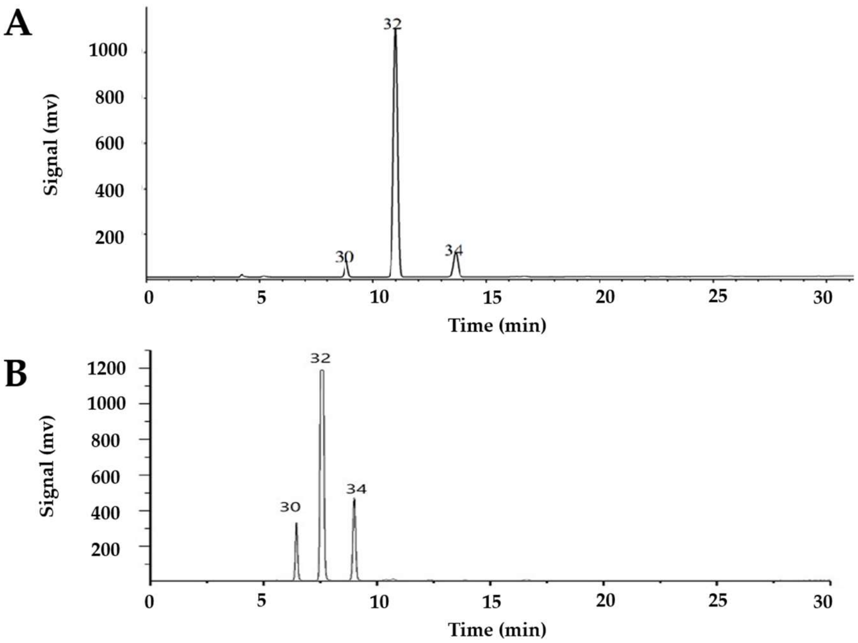

Figure S1. High performance liquid chromatography (HPLC) profile of CCSKO obtained by hot-pressing (A) and aqueous enzyme extraction (B) methods, respectively.

Table S1. L5178Y cell TK gene mutation frequency induced by CCSKO.

(A) L5178Y cell TK gene mutation frequency (-S9) induced by CCSKO.

Group	Dose (μg/mL)	T-MF ($\times 10^{-6}$)	S-MF ($\times 10^{-6}$)	L-MF ($\times 10^{-6}$)	SCM (%)
High	5000	83.75 ± 11.44^{a}	79.68 ± 5.69^{a}	36.83 ± 10.69^{a}	95.57 ± 6.27^{a}
Medium	2500	76.79 ± 10.49^{a}	73.15 ± 15.6^{a}	23.43 ± 4.82^{a}	94.75 ± 7.43^{a}
Low	1250	59.08 ± 4.22^{b}	53.15 ± 4.17^{ab}	16.19 ± 0.00^{b}	89.94 ± 0.64^{a}
NC	1%	87.47 ± 4.95^{a}	77.10 ± 9.72^{a}	21.44 ± 4.41^{a}	87.97 ± 6.13^{a}
MMS	5	851.44 ± 36.91^{c}	838.26 ± 18.2^{c}	229.79 ± 11.46^{c}	98.50 ± 2.12^{a}
Menstruum	1%	61.74 ± 7.78^{ab}	58.97 ± 3.87^{b}	22.19 ± 3.57^{a}	95.88 ± 5.82^{a}

(B) L5178Y cell TK gene mutation frequency (+S9) induced by CCSKO.

Group	Dose (μg/mL)	T-MF (×10^{-6})	S-MF (×10^{-6})	L-MF (×10^{-6})	SCM (%)
High	5000	89.16 ± 10.44[a]	81.82 ± 10.31[a]	29.41 ± 4.73[a]	91.72 ± 0.83[a]
Medium	2500	88.67 ± 12.12[a]	75.89 ± 5.95[a]	23.12 ± 0.00[b]	86.86 ± 18.58[a]
Low	1250	61.63 ± 9.21[b]	58.43 ± 13.73[a]	26.89 ± 4.33[a]	94.20 ± 8.21[a]
NC	1%	54.36 ± 8.12[b]	51.47 ± 4.04[ab]	21.05 ± 7.59[a]	95.19 ± 6.80[a]
CP	15	355.81 ± 8.27[d]	294.52 ± 15.05[d]	106.33 ± 5.63[c]	82.85 ± 6.16[a]
Menstruum	1%	73.12 ± 3.87[bc]	64.97 ± 0.00[abc]	21.44 ± 3.45[a]	88.98 ± 4.71[a]

T-MF, total colonies mutation frequency; S-MF, small colonies mutation frequency; L-MF, large colonies mutation frequency; MMS, methyl methane sulfonate; CP, cyclophosphamide; NC, negative control group.

Data are expressed as mean ± SD; Values with different letters in the same column indicate significant differences ($p < 0.05$).

Table S2. The cell toxicity in *in vitro* mammalian cell TK gene mutation test of CCSKO.

Group	Dose (μg/mL)	PE_0 (%)		PE_2 (%)		RS0 (%)		RS2 (%)		RSG (%)		RTG (%)	
		-S9	+S9	-S9	+S9	-S9	+S9	-S9	+S9	-S9	+S9	-S9	+S9
High	5000	79.28	74.82	72.70	81.64	88.78	76.31	67.19	72.90	93.83	81.61	63.04	59.50
Medium	2500	94.99	72.70	79.28	68.66	106.37	74.15	73.28	61.31	92.59	89.66	67.85	54.97
Low	1250	74.82	77.01	98.04	89.30	83.78	78.55	90.61	79.75	93.83	94.25	85.02	75.16
NC	1%	89.30	98.04	86.64	101.24	100.00	100.00	80.08	90.41	100.00	80.46	80.08	72.74
MMS	5	32.58		56.30		36.46		52.03		43.21		22.48	
CP	15		38.32		77.01		39.09		68.77		51.72		35.57
Menstruum (DMSO)	1%	92.08	98.04	108.20	111.98	103.11	100.00	100.00	100.00	100.00	100.00	100.00	100.00

PCE, polychromatic erythrocytes; NCE, normochromatic erythrocyte.

References

1. Siger A.; Józefiak M. The effects of roasting and seed moisture on the phenolic compound levels in cold-pressed and hot-pressed rapeseed oil. *Eur. J. Lipid Sci. Technol*. **2016**, 118, 1952-1958. https://doi.org/10.1002/ejlt.201500249.
2. Zeng C.; Zhao R.; Ma M.; Zeng Z.; Gong D. Mutagenesis and chararcterization of a *Bacillus amyloliquefaciens* strain for Cinnamomum camphora seed kernel oil extraction by aqueous enzymatic method. *AMB Expr*. **2017**, 7, 154. https://doi.org/10.1186/s13568-017-0451-9.
3. OECD. Test No. 423: Acute oral toxicity - Acute toxic class method. **2002**. https://doi.org/10.1787/9789264071001-en.
4. OECD. Test No. 474: Mammalian erythrocyte micronucleus test. **2016**. https://doi.org/10.1787/9789264264762-en.
5. OECD. Test No. 471: Bacterial reverse mutation test. **2020**. https://doi.org/10.1787/9789264071247-en.
6. OECD. Test No. 490: *In Vitro* mammalian cell gene mutation tests using the thymidine kinase gene. **2016**. https://doi.org/10.1787/9789264264908-en.
7. AOCS. Official Method Ce 2-66: Preparation of methyl esters of fatty acids. In M. W. Collison, (Ed.), *Official methods and recommended practices of the AOCS (7th ed.)*. **2017**, Urbana, IL: AOCS Press.
8. AOCS. Official Method Ce 1-62: Fatty acid composition by gas chromatography. In M. W. Collison, (Ed.), *Official methods and recommended practices of the AOCS (7th ed.)*. **2017**, Urbana, IL: AOCS Press.
9. Sun C.; Wei W.; Su H.; Zou X.; Wang X. Evaluation of *sn*-2 fatty acid composition in commercial infant formulas on the Chinese market: A comparative study based on fat source and stage. *Food Chem*. **2018**, 242, 29-36. https://doi.org/10.1016/j.foodchem.2019.09.005.
10. AOCS. Official Method Ce 5b-89: Triglycerides in Vegetable Oils by HPLC. In M. W. Collison, (Ed.), *Official methods and recommended practices of the AOCS (7th ed.)*. **2017**, Urbana, IL: AOCS Press.
11. Uncu O.; Ozen B.; Tokatli F. Use of FTIR and UV- visible spectroscopy in determination of chemical characteristics of olive oils. *Talanta*. **2019**, 201, 65-73. https://doi.org/10.1016/j.talanta.2019.03.116.
12. Yan X.; Liang S.; Peng T.; Zhang G.; Zeng Z.; Yu P.; Gong D.; Deng S. Influence of phenolic compounds on physicochemical and functional properties of protein isolate from *Cinnamomum camphora* seed kernel. *Food Hydrocoll*. **2020**, 102, 105612. https://doi.org/10.1016/j.foodhyd.2019.105612.
13. AOCS. Official Method Cc 7-25: Refractive index of fats and oils. In M. W. Collison, (Ed.), *Official methods and recommended practices of the AOCS (7th ed.)*. **2017**, Urbana, IL: AOCS Press.
14. AOCS. Official Method Cc 13b-45: Color of fats and oils, Lovibond (Wesson) Wesson method using color glasses calibrated in accordance with the AOCS-tintometer color scale. In M. W. Collison, (Ed.), *Official methods and recommended practices of the AOCS (7th ed.)*. **2017**, Urbana, IL: AOCS Press.
15. AOCS. Official Method Ca 2f-93: Moisture and Volatile Matter in Fats and Oils, Modified Method. In M. W. Collison, (Ed.), *Official methods and recommended practices of the AOCS (7th ed.)*. **2017**, Urbana, IL: AOCS Press.
16. AOCS. Official Method Cd 3d-63: Acid value. In M. W. Collison, (Ed.), *Official methods and recommended practices of the AOCS (7th ed.)*. **2017**, Urbana, IL: AOCS Press.
17. AOCS. Official Method Cd 3-25: Saponification value. In M. W. Collison, (Ed.), *Official methods and recommended practices of the AOCS (7th ed.)*. **2017**, Urbana, IL: AOCS Press.
18. AOCS. Official Method Cd 1b-87: Iodine value of fats and oils cyclohexane method. In M. W. Collison, (Ed.), *Official methods and recommended practices of the AOCS (7th ed.)*. **2017**, Urbana, IL: AOCS Press.
19. AOCS. Official Method Cd 8b-90: Peroxide value acetic acid-isooctane method. In M. W. Collison, (Ed.), *Official methods and recommended practices of the AOCS (7th ed.)*. **2017**, Urbana, IL: AOCS Press.
20. AOCS. Recommended practice Aa 11-05: Aflatoxins by HPLC using postcolumn photochemical derivatization. In M. W. Collison, (Ed.), *Official methods and recommended practices of the AOCS (7th ed.)*. **2017**, Urbana, IL: AOCS Press.
